# Nifedipine-Influenced Enlargement of the Masticatory Mucosa in an Elderly Edentulous Patient: A Rare Case Report with a Two-Year Follow-Up

**DOI:** 10.1155/2024/6889574

**Published:** 2024-03-28

**Authors:** Shoufu Sun, Yufan Pan, Jichun Zhang, Yunan Jiang

**Affiliations:** ^1^Department of Stomatology, Tongren Hospital, Shanghai Jiaotong University School of Medicine, 1111 Xianxia Road, Shanghai 200336, China; ^2^Xianxia Community Care Center, 140 Furong River Road, Shanghai 200336, China

## Abstract

Drug-influenced gingival enlargement is a common side effect associated with certain medications, particularly calcium channel blockers like nifedipine, which has been extensively documented. However, the occurrence of nifedipine-influenced masticatory mucosa overgrowth in edentulous patients is rare. Here, we present a case of nifedipine-influenced mucosal enlargement persisting in a 67-year-old edentulous patient 3 months after the extraction of all his teeth. The patient underwent flap surgery and alveoloplasty to excise the overgrown tissue, followed by complete denture restoration. The antihypertensive medication was replaced with valsartan. A 2-year follow-up revealed no recurrence of overgrowth, indicating the effectiveness of this management strategy for such clinical situation.

## 1. Introduction

Drug-influenced gingival enlargement refers to the aesthetically disfiguring overgrowth of the gingiva associated with nondental medications [1]. These lesions typically manifest in the anterior region, often initially appearing in the gingival papilla. The first reported case series documenting drug-influenced gingival enlargement traced back to 1939, revealing that 68 out of 119 patients treated with phenytoin, an antiepileptic drug, developed gingival hyperplasia [2]. Subsequent studies have extensively demonstrated that the side effects of various drugs, including antiepileptic drugs (e g., phenytoin), immunosuppressant (e g., cyclosporine A), and calcium channel blockers (e g., nifedipine), can lead to gingival overgrowth [1, 3, 4].

Currently, calcium channel blockers (CCBs) like nifedipine, felodipine, amlodipine, nitrendipine, and diltiazem are widely used in the clinical management of hypertension [5]. The first case of CCB-induced gingival enlargement was introduced in 1984 [6]. Subsequent clinical reports have indicated that nifedipine poses a higher risk of gingival overgrowth compared to other CCBs such as amlodipine, with its prevalence ranged between 6.3% and 83% [7–10]. While gingival overgrowth associated with various drugs has been well documented, drug-influenced enlargement of the masticatory mucosa in edentulous individuals has rarely been observed. Most cases of mucosal enlargement in edentulous patients have been linked to phenytoin or cyclosporin intake [11–15], with only a few instances reporting nifedipine medication [16]. Here, we present a case of nifedipine-influenced masticatory mucosa enlargement in an edentulous male, focusing on both the clinical management the long-term efficacy of the treatment.

## 2. Case Report

A 67-year-old male was referred to the Dental Outpatient Department of Tongren Hospital, Shanghai Jiao Tong University School of Medicine, with a chief complaint of “missing teeth and swollen gums for 3 months”. The patient had been in a partially edentulous state for several years without receiving any dental restoration. Three months prior to the visit, all remaining teeth were severely loose and subsequently extracted due to “chronic periodontitis”. Notably, the patient reported swelling and hyperplasia of the gingival papilla observed before the tooth extraction. Seeking complete denture restoration, the patient discovered that despite the extractions, his gums remained severely swollen, affecting both edentulous jaws.

**Figure 1:**
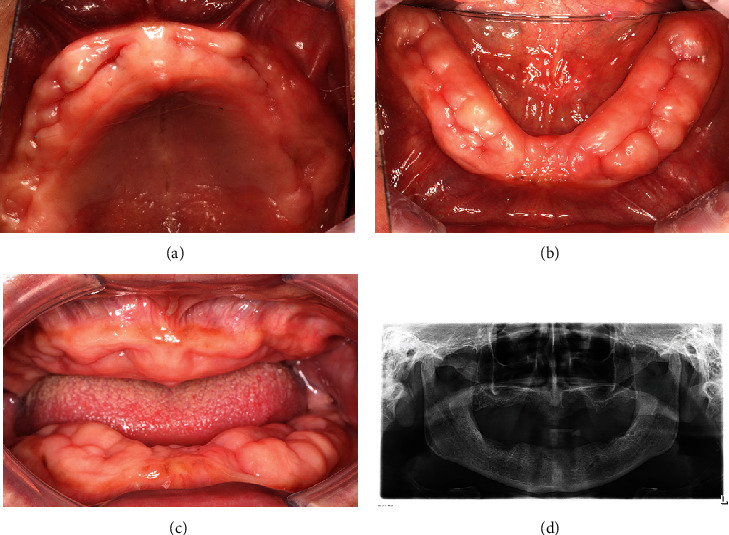
Intraoral images before surgery: (a) The occlusal view of maxillary alveolar ridge; (b) the occlusal view of mandibular alveolar ridge; (c) the frontal view of the edentulous ridges; and (d) the panoramic imaging.

The patient had a three-year history of hypertension and was prescribed a drug regimen of 10 mg of nifedipine twice daily. Blood glucose level and blood pressure were within normal ranges, and routine blood test revealed normal hemoglobin, red and white blood cell count, platelet count, bleeding time, and clotting time. Upon oral examination, the patient presented with a reduced lower facial height and complete edentulous maxilla and mandible. Mucosal enlargement was evident on both edentulous ridges, displaying a pale-pink, firm, irregular, mulberry-shaped, and nodular appearance (Figure 1(a)–1(c)). The keratinized mucosa was wide and did not tend to bleed upon probing. Additionally, panoramic radiograph showed unevenly absorbed maxillary and mandibular ridges with no osseous deformities (Figure 1(d)).

**Figure 2:**
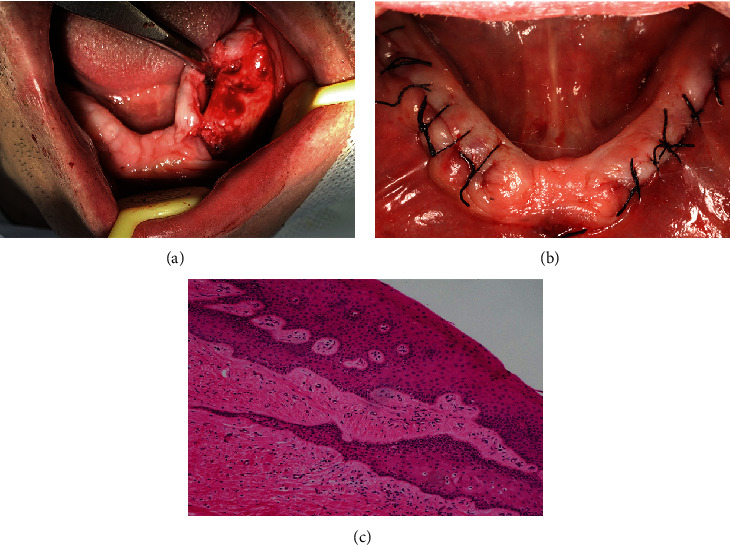
Intraoperative and postoperative images: (a) intraoperative flap, considerable granulation tissue with uneven alveolar bone; (b) the wound closure and suture postoperatively; and (c) the pathological section showed that hyperplastic epithelium overlay fibrous connective tissue.

Upon medical approval, preprosthetic surgeries including flap surgery and alveoloplasty were performed (Figures 2(a) and 2(b)). Before surgery, the patient rinsed his mouth with 0.12% chlorhexidine gluconate solution as an antiseptic mouthwash for 60 seconds. For oral and maxillofacial disinfection, iodophor (Lanso, skin mucosa disinfectant, 60 ml, active ingredients and content: iodine, effective iodine content (*w*/*v*) 0.45%-0.55%, and chlorhexidine acetate content (*w*/*v*) 0.028%-0.034%) was utilized. After routine draping, local anesthesia was performed using 4% articaine (10 ml) with 1/100,000 adrenaline. An incision with no. 11 blade was made on the crest of the alveolar ridge, extending from the anterior region to the posterior molar region. Releasing incisions were made on both the mesially and distally labial sides, and the flap was turned to the bone surface. Hypertrophy tissues were excised, with partial keratinized mucosa retained, and granulation tissues were carefully scraped off. Both alveolar bone surfaces underwent trimming and reshaping using a turbine and a bone file. An incisional biopsy was obtained for pathological evaluation, revealing hyperplastic epithelium overlaying fibrous connective tissue, leading to a fibrous epulis pathological diagnosis (Figure 2(c)). Simple interrupted sutures with 4-0 silk were applied on the alveolar ridge thread for surgical closure. The prescribed postoperative care included systemic antibiotics (cefradine capsule 250 mg q6h for 3 days), anti-inflammatory analgesic drugs (compound paracetamol tablet 400 mg per day for 3 days), and a 0.2% chlorhexidine mouthwash (twice a day for 7 days). The mandibular surgery was conducted one month after the maxillary surgery. Postoperatively, the patient reported no severe discomforts or complications. Following the oral interventions, the patient consulted his cardiologist, leading to a replacement of the antihypertensive medication with valsartan 80 mg per day.

**Figure 3:**
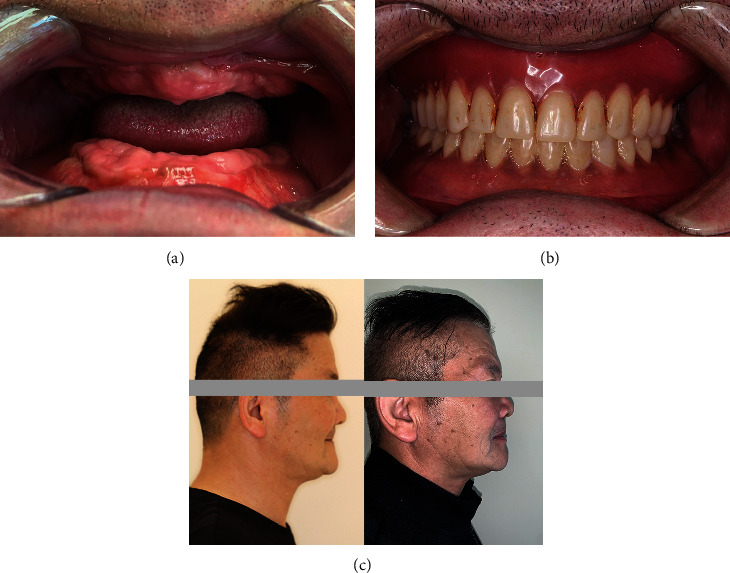
(a) Intraoral images 2 months after surgery; (b) complete denture restoration; and (c) lateral photos showing significantly improved profile after denture restoration.

**Figure 4:**
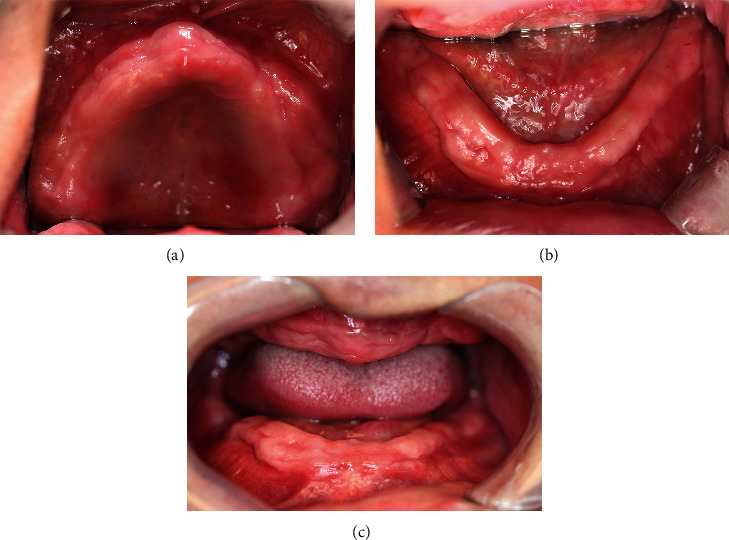
Intraoral images 18 months after surgery: (a) the occlusal view of the maxillary alveolar ridge; (b) the occlusal view of the mandibular alveolar ridge; and (c) the frontal view of the edentulous ridges.

After a 2-month healing period, the patient's alveolar ridges displayed a firm, regular, and well-healed appearance with no signs of recurrence or overgrowth (Figure 3(a)). Subsequently, complete denture fabrication for both arches were accomplished (Figure 3(b)), restoring the patient's masticatory function as well as enhancing lower facial aesthetic (Figure 3(c)). The patient underwent recall appointment at 18 months and 24 months after surgery, during which no recurrence of overgrowth was observed (Figure 4, Supplementary Figure 1). His dentures fitted well with good retention and aesthetic effect.

## 3. Discussion

Drug-influenced gingival enlargement typically manifests within the first 3 months of drug administration. The overgrowth initially affects the interdental gingival papilla and then extends to the facial and lingual gingival margin, involving attached gingiva [17]. This enlargement can lead to malposition of the teeth, impaired masticatory function, and compromised oral hygiene. Various risk factors, including plaque, age, gender, and drug variables, have been identified [3]. Among these, plaque is a significant cofactor in the progression of drug-influenced gingival overgrowth and is even considered as a prerequisite [18]. In the presence of plaque accumulation, the hyperplastic gingiva appears large, lobulated, and soft and has an obvious tendency to bleed, whereas in the absence of inflammation, swollen gums are pale-pink, firm in texture, and do not bleed on probing [7]. In the presented case, the patient experienced tooth loss attributed to chronic periodontitis. Notably, gingival overgrowth occurred before tooth extraction, underscoring the importance of good oral hygiene and effective plaque control perioperatively and in the long run. Typically, gingival enlargement caused by inflammation tends to resolve after tooth extraction, and the enlargement of the masticatory mucosa in an edentulous ridge rarely occurs [16]. However, in this case, the patient observed that his enlarged gums did not recover, instead, the condition worsened postextraction. Given the absence of local irritation factors, a thorough examination of the patient's medical history revealed a 3-year history of hypertension with nifedipine medication. By integrating clinical history, intraoral manifestations, and a fibrous epulis pathological diagnosis, a conclusive diagnosis of nifedipine-influenced enlargement of the masticatory mucosa was established. Previously, most reports concerning drug-influenced mucosa hyperplasia in edentulous patients were associated with phenytoin use [11, 12, 15, 19, 20]. Thomason et al. reported a case of severe mucosal hyperplasia in the edentulous maxilla associated with immunosuppressant therapy [13]. In recent years, Asif et al. described an edentulous patient suffering from nifedipine-influenced gingival enlargement and took surgical excise [16], and Mathur et al. presented a similar case associate with amlodipine intake [21]. In our report, we presented a rare case of gingival enlargement associated with nifedipine, persisting in an edentulous state, emphasizing the need of extra attention to be paid to edentulous patients undergoing antihypertensive therapy simultaneously.

The primary histopathological features of drug-influenced gingival enlargement include thickening epithelium, predominant fibrotic changes in the connective tissue, an accumulation of extracellular matrix, and varying degrees of inflammation [22]. The epithelial layer exhibits elongated rete ridges and hyperplastic and acantholytic stratified squamous epithelium [16]. In the underlying connective tissue, there is an increased number of active fibroblasts, abundant collagen fiber bundles, and various amorphous substances interspersed among the chronic inflammatory cells, mainly plasma cells, which accumulate in areas with newly formed capillaries [11, 16, 23]. In our presented case, pathological analysis revealed a diagnosis of fibrous epulis characterized by hyperplastic epithelium with rete ridges extending into the connective tissue and abundant collagen fiber bundles in the lamina propria, which was similar to the findings of previous reports. Also, inflammatory cells are presented around the capillaries, yet the inflammation was at low grade. This histopathological diagnosis aligns with the clinical findings observed.

Gingival enlargement is associated with an imbalance between the synthesis and degradation of the extracellular matrix, including collagen fibers [24]. Gingival fibroblasts, which constitute the most abundant cell population in the gingival connective tissue, may contribute to mucosal enlargement in edentulous patients through the incorporation of specific populations of fibroblasts [16]. The elevation of various growth factors by drugs, such as tumor growth factor (TGF)-*β*1, a key mediator of epithelial-mesenchymal transition, facilitates the epithelial cells to assume the mesenchymal cell phenotype (fibrogenic fibroblast-like cells) [23]. Additionally, drugs impact intracellular calcium homeostasis, leading to changes in the activity of matrix metalloproteinases and the failure to activate collagenase [23]. When collagen production by fibroblasts increases, while degradation and intracellular digestion decrease, an excess of collagen tends to accumulate [25].

The management strategies of gingival overgrowth encompass both nonsurgical and surgical approaches. In the realm of nonsurgical interventions, the primary objective is to reduce gingival inflammation, thereby mitigating the necessity for surgery. Hancock and Swan proposed that significant reduction of nifedipine-influenced gingival overgrowth could be achieved through thorough scaling and root planning coupled with meticulous plaque control [26]. While the efficacy of oral hygiene therapy remains to be further confirmed, it is undoubted that patients undergoing gingival overgrowth stand to benefit from effective plaque control. Antiseptic mouthwashes, such as chlorhexidine solution, have been employed for adjunctive chemical plaque removal. However, no evidence suggests that these mouthwashes can help reduce gingival overgrowth or prevent recurrence after surgery [27]. Some reports indicate that systemic antibiotics (e.g., azithromycin and metronidazole) may be beneficial in managing gingival overgrowth, with their effects potentially attributed to the reduction of bacterial infection and inflammation [28, 29]. It is noteworthy that azithromycin could also enhance the phagocytic activity of gingival fibroblasts in rats, thereby increase collagen degradation [30]. Another crucial strategy for addressing drug-influenced gingival enlargement is changing the specific medication. In the case of CCBs, various alternatives are available to achieve similar antihypertensive efficacy. In our presented case, nifedipine was replaced by valsartan to mitigate the possibility of recurrence after surgery, and no mucosal enlargement was observed in the 2-year follow-up.

Despite all these nonsurgical treatment attempts, surgical management remains the most common approach for overgrown gingiva or mucosa, encompassing techniques such as gingivectomy, flap surgery, electrosurgery, and laser excision [27]. Gingivectomy was first introduced to the management of drug-influenced gingival overgrowth by Thompson and Gillespie in 1941 [31]. On the other hand, flap surgery may be advocated for patients of mild-to-moderate overgrowth accompanied by both bone and attachment loss. In the presented case, alveoloplasty was also performed to facilitate subsequent full denture restorations. Perioperative hemorrhage is the main disadvantage, especially when the gingiva tissue was inflamed and highly vascularized [27]. Electrosurgery and laser are both widely used alternatives to conventional scalpel gingivectomy and might be advantageous in cases with impaired hemostasis, although reports had confirmed a delayed wound healing compared to scalpel gingivectomy [32].

In conclusion, drug-influenced mucosa enlargement in edentulous patients rarely happens clinically, especially in association with nifedipine. The etiology might be attribute to specific fibroblast subpopulations, yet remains to be further elucidated. Flap surgery is an effective and stable management for this case, and an alternative medication choice made by physicians shall be crucial to avoid long-term recurrence.

## Data Availability

All data related to the presented case are included within the article and the supplementary files.

## Supplementary Materials

Supplementary Materials Supplementary Figure 1: the intraoral images of the maxillary (A) and mandibular (B) edentulous ridges 24 months after surgery.
