# Supplementary figures and images for: Nifedipine-Influenced Enlargement of the Masticatory Mucosa in an Elderly Edentulous Patient: A Rare Case Report with a Two-Year Follow-Up
Source: Case Rep Dent. 2024 Mar 28;2024:6889574. doi: 10.1155/2024/6889574 (PMC10994707; doi:10.1155/2024/6889574)

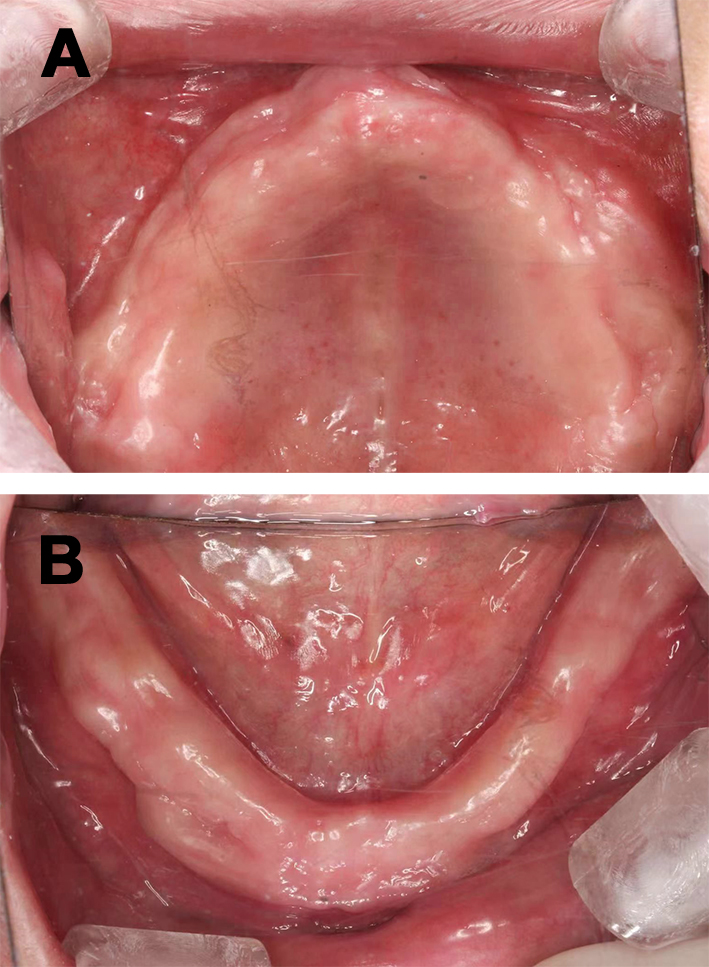

Supplement: Supplementary Materials — Supplementary Figure 1: the intraoral images of the maxillary (A) and mandibular (B) edentulous ridges 24 months after surgery. [file 6889574.f1.jpg]
